# Facing COVID-19 Pandemic in a Tertiary Hospital in Milan: Prevalence of Burnout in Nursing Staff Working in Sub-Intensive Care Units

**DOI:** 10.3390/ijerph18136684

**Published:** 2021-06-22

**Authors:** Alberto Bisesti, Andrea Mallardo, Simone Gambazza, Filippo Binda, Alessandro Galazzi, Silvia Pazzaglia, Dario Laquintana

**Affiliations:** Healthcare Professions Department, Fondazione IRCCS Ca’ Granda Ospedale Maggiore Policlinico, Via Francesco Sforza 35, 20122 Milano, Italy; alberto.bisesti@policlinico.mi.it (A.B.); andrea.mallardo@policlinico.mi.it (A.M.); simone.gambazza@policlinico.mi.it (S.G.); filippo.binda@policlinico.mi.it (F.B.); silvia.pazzaglia@policlinico.mi.it (S.P.); dario.laquintana@policlinico.mi.it (D.L.)

**Keywords:** burnout, COVID-19, healthcare workers, nurses, stress

## Abstract

In early March 2020, Italy became the epicenter of the Coronavirus Disease 2019 (COVID-19) pandemic in Europe. A different organization of hospital units was required to take care of patients affected by acute respiratory failure caused by COVID-19. This study aimed to evaluate the prevalence of burnout in two sub-intensive care units (SICUs) of the COVID-19 hub center of the Lombardia region in Milan (Italy). All nurses and healthcare assistants working in the SICUs during June 2020 were included in the study. Burnout was assessed via the Maslach Burnout Inventory questionnaire. One hundred and five (84%) SICU staff participated in the study. The prevalence of high burnout for nurses and healthcare assistants was 61.9% for emotional exhaustion, 47.6% for depersonalization and 34.3% for personal accomplishment. Depersonalization was significantly more frequent in younger nurses (*p* = 0.009). Nurses were 4.5 times more likely to have burnout than healthcare assistants. Burnout was a common condition among healthcare workers operating in SICUs during the pandemic. Urgent actions are needed, especially for nurses, as well as preventive strategies for future pandemic scenarios.

## 1. Introduction

At the end of 2019, Severe Acute Respiratory Syndrome Coronavirus 2 (SARS-CoV-2) was recognized as the causative agent of a cluster of severe pneumonia cases in Hubei Province in China [1]. Italy was the first western country facing an outbreak of Coronavirus Disease 2019 (COVID-19). The first patient diagnosed with COVID-19 was admitted on 20 February 2020 to the intensive care unit (ICU) in Codogno hospital (Lombardia, Italy), and the number of reported positive cases increased exponentially afterwards [2]. The infection spread rapidly worldwide, and on 11 March 2020 COVID-19 was defined by the World Health Organization as a pandemic [3].

Under this scenario, several hospital wards in Lombardia, the most affected northern region in Italy, had to be converted into COVID-19 sub-intensive units (SICUs) [4] in order to enlarge their capacity to take care of patients requiring non-invasive ventilatory support, not manageable any longer in saturated ICUs.

Several professionals were involved in the care of patients suffering from acute respiratory failure caused by SARS-CoV-2 pneumonia [4]. Hospital staff were necessarily implemented and new professionals were rapidly recruited in order to guarantee adequate assistance [5], avoiding high workload-to-staffing ratio. Intuitively, the impact of COVID-19 on the psychological wellbeing of healthcare workers (HCW) was remarkable [6]. Particularly, nurses reported high levels of work-related stress and burnout [7,8], just as occurred during the SARS-CoV epidemic of 2002–2004 [9]. Burnout, defined by emotional exhaustion (EE), depersonalization (DP) and personal accomplishment (PA), is known to detract from optimal working capacities, and has been found to be driven by high job stress, high time pressure and workload, and poor organizational support [10]. Recently, an Italian study evaluated the mental health of HCWs involved in the care of patients in highly affected COVID-19 areas [11]. The percentages of those who reported mental problems significantly differed by occupational profile, with nurses being at higher risk of developing symptoms of post-traumatic distress and anxiety. Staff working in SICUs and ICUs reported more traumatic events than staff working in COVID-19-free units [11]. Overall, larger than 50% prevalence of burnout risk among HCWs working in COVID-19 units was also reported by other international studies [12,13,14,15].

SICUs have rapidly become the new ward paradigm of our COVID-19 hub center, with ICUs being saturated by patients, often heavily sedated. As an intermediate unit between the emergency department and ICU, the number of nurses and healthcare assistants in SICUs was the first to be increased, with the consequences of exposing untrained and young professionals to a high burden, in terms of working hours, assistance demands and mental health.

The aim of the present study is to evaluate the prevalence of burnout syndrome in nurses and healthcare assistants operating in the COVID-19 SICUs of our institution in Milan, Italy.

## 2. Materials and Methods

A prevalence study was carried out in June 2020 at the Fondazione IRCCS Ca’ Granda Ospedale Maggiore Policlinico, identified as one of the COVID-19 hub centers for the Lombardia region.

### 2.1. Staff and Organization of SICUs

During the first days of the pandemic, the high dependency unit (43 beds) and the pneumology unit (40 beds) were reconverted from a logistical point of view and equipped with the necessary technologies for patient monitoring. In total, the two SICUs had 83 beds and were structured in a similar way: a filter area (called green zone) was created and included changing rooms, a warehouse storage area, a dressing area with all the personal protective equipment (PPE), and the area where the patients stayed (called red zone). The staff-to-patient ratio was raised up to 1:5 for nurses and 1:12 for healthcare assistants. The staff consisted of both low and highly experienced professionals. In order to optimize the delivered care, 35% of working teams consisted of highly skilled professionals. Before the opening of the COVID-19 SICUs, short training days were offered, with the purpose of educating the employees about the correct use of PPE and about the procedures and protocols to be followed.

The two SICUs accepted their first patient at the beginning of March 2020 and closed during the middle of June, when the contagion wave flattened in Italy. Generally, hospitalized patients required oxygen therapy via a reservoir mask, Venturi mask, nasal cannula (19%), or high flow nasal cannula (14%) and continuous positive airway pressure ventilation (67%).

### 2.2. Measures

The Maslach Burnout Inventory (MBI) questionnaire [16] adapted to healthcare workers (MBI-Human Services Survey) [17] was used to assess burnout level. The questionnaire consists of 22 items exploring the intensity and frequency of effects, symptoms and emotional perceptions related to work. The items measure three independent dimensions of burnout: 9 items for EE, 5 items for DP and 8 items for PA. EE represents the loss of enthusiasm for work, with a sense of emptiness and impaired emotional resources to face habitual workload; DP consists of negative feelings of cynicism and indifference towards patients and even colleagues; and PA consists of a self-perception of extreme inadequacy at work and a decline in professional competences.

Responses are based on a seven-point Likert scale, ranging from 0 (never) to 6 (every day). Higher mean scores for DP and EE subscales and lower mean scores on the PA subscale correspond to a higher degree of burnout. The considered ranges to define high burnout were EE ≥ 24, DP ≥ 9 and PA ≥ 37. We considered a meaningful manifestation of burnout if at least one subscore was above these cut-offs.

The MBI questionnaire was administered online using the free platform SURVIO^®^ an online tool for data collection and analysis. A customized e-mail was sent to all nurses and healthcare assistants, inviting them to fill in the questionnaire on a voluntary basis. The MBI questionnaire was self-administered, and all participants accepted the informed consent form. Demographic characteristics (i.e., age, sex), job and years of work experience were collected as well and processed anonymously.

### 2.3. Statistical Analysis

Variables were summarized by mean and standard deviation or count and percentage. Prevalence of burnout was reported as proportion with 95% confidence interval. Burnout levels were compared across professional roles by the means of Chi-squared or Fisher exact test, as appropriate. Wilcoxon rank-sum test was used to compare MBI scoring to the Italian normative reference sample [17]. The correlation between age, EE, DP and PA was described by Spearman’s correlation coefficient and the Benjamini–Hochberg method was applied to correct for multiple testing. A logistic model was further fitted to capture the statistical association between burnout and independent variables. All analyses were performed using R Core Team (version 4.0.3).

## 3. Results

A total of 105 out of 125 (84%) staff working in SICUs completed the questionnaire. Considering the whole population of nurses and healthcare assistants employed in the SICUs, the sample included 87 (90.6%) registered nurses and 18 (62.1%) healthcare assistants. Despite the fact that we used convenience sampling, a study of this size, with a desired significance of 0.05 and a desired power of 0.90, can reliably detect an effect size of about 0.32 (Cohen’s d). The participants’ characteristics are shown in Table 1. On average, nurses were 5.9 years younger than healthcare assistants (*p* = 0.027).

Overall, estimates for EE, DP and PA were 29.1 (12.7), 9 (5.8) and 32.1 (7.8), respectively, with 83 participants (79%, 95% CI 69.8 to 86.1) having a high likeliness of burnout, many of them from the nursing staff (69.5%, 73/105). The prevalence of high burnout in the three dimensions was 61.9% for EE, 47.6% for DP and 34.3% for PA. The prevalence of the other levels of burnout is reported in Table 2.

A comparison of our findings with normative values available for Italian healthcare staff showed that average EE and DP scoring was 8.9 and 1.97 points higher (*p* < 0.001), respectively. No evidence of difference was detected when comparing PA (*p* = 0.607).

A statistically significant association was found between the presence of burnout and the professionals involved in the present analysis (*p* = 0.0177). Particularly, levels of burnout also remained associated with the type of activity performed in SICUs in the EE (*p* = 0.016) and DP dimension (*p* = 0.017). The type of profession was not associated with levels of burnout in the PA dimension (*p* = 0.753).

**Figure 1:**
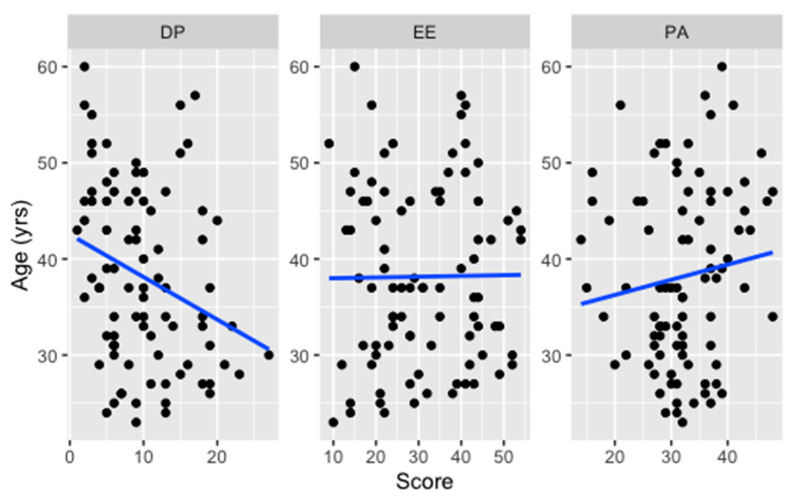
Correlation (solid line) between age and DP, EE and PA scores among nurses.

Looking at the relationship between age and the total score of DP (Figure 1), a weak negative correlation was found in nurses (rho= −0.28, *p* = 0.009) and healthcare assistants (rho= −0.31, *p* = 0.196).

Correlation between age and PA was also weak but diverging for nurses (rho= 0.13, *p* = 0.249) and healthcare assistants (rho= −0.27, *p* = 0.264). Eventually, correlation between age and EE revealed a higher positive association in healthcare assistants (rho= 0.45, *p* = 0.061) than nurses (rho= 0.01, *p* = 0.924).

From the fitted logistic regression model, nurses were 4.5 times more likely to have burnout than healthcare assistants, after adjusting for age, sex and career length. However, these independent variables explained only 10% of the total variance. The same model adjusted for the job location (i.e., SICU) did not yield different results.

## 4. Discussion

The present survey was carried out in a very troubled and unprecedented period; however, we achieved a high response rate, maybe indicating that staff were in need of communicating on this topic. Overall, our findings show that almost the 80% of the participants have a high probability of developing burnout, nurses in particular. More than half of the professionals involved (61.9%) show high levels of EE, and nearly half of participants show high levels of DP (47.6%), whereas almost one third show low PA. These data express a worrisome situation for the wellbeing of the HCWs employed in our SICUs and, by consequence, may constitute a barrier to the quality of assistance delivered to patients.

This high risk of burnout has been highlighted in recent studies concerning the association between burnout and the COVID-19 pandemic in HCWs involved in the front line in the care of patients with COVID-19 all over the world [18,19,20,21,22]. In the scores achieved by our participants, EE, DP and PA are higher than the scores obtained in two similar studies also conducted in one hospital in the north of Italy [11,23], and double compared to those from another Italian study carried out by Barello et al. [24]. As reported by Azoulay et al. [15], COVID-19 alone could explain the large prevalence of burnout among HCWs, much more than we could have expected.

Indeed, levels of burnout—except for professional accomplishment—in our study were found to be higher compared to those from the Italian study for the MBI validation [17]. These results are intuitively understandable, considering that the high mortality of COVID-19 could have negatively affected the level of DP and EE. The working environment played a role in mediating the emotions of HCWs. Once on duty, HCWs feared developing SARS-CoV-2 infection as well as carrying the virus home, thus infecting their families and friends [25]. At the same time, HCWs had to face the limited resources and information available at the time of the first wave, such as the widespread unavailability of PPE or surgical masks. Additionally, the lack of agreement among healthcare organizations regarding whether surgical masks or N95 respirators were effective did not help in the control of fear [26,27]. To this, one should add that the absence of a penal shield towards negligence allegations [28] has exposed HCWs to further stress and fear. On the contrary, the low rate of HCWs infected by SARS-CoV-2 in our institution [29,30] and the absence of victims among the staff could have had a mitigating effect.

With regard to personal accomplishment, we reported lower scores compared to those from the Italian study [17], without reaching the statistical threshold.

The present findings also reveal an association between burnout and job. Nurses were 4.5 times more likely to develop burnout than healthcare assistants. This could be likely due to the fact that nurses had the most contact with patients with COVID-19 and they were directly at risk when performing treatments and care [31]. Moreover, younger nurses showed higher levels of DP, which tend to decrease with the age of participants. Lack of experience, new and complex work realities, both technically and relationally, and the distance or the isolation from family (which helps to cope with work stress) may partly explain why younger nurses reported higher levels of burnout.

As reported by other studies, nurses with a lower age and less work experience show higher levels of burnout than nurses with more work experience and older nurses [19,32]. However, just as it has happened during these months, hiring and assigning new staff to the wards most in need, i.e., SICUs and ICUs, is inevitable. Any forms of professional resilience should therefore be expected.

To better deal with the pandemic, hospitals have undergone profound organizational changes [33,34]. Among the measures adopted by hospitals to reduce cross-infections, there was a ban on visits to patients by family members [35]. These restrictions generated conflicting emotions among HCWs, especially during end-of-life situations [36,37]. Some of the personnel involved in our study were reallocated from other wards and had to change their way of working and their perception towards disease threat. The study carried out by Catania et al. shows that stress in nurses was increased by shift patterns and by working hours that changed regularly, creating emotional stress and uncertainty regarding the workplace and colleagues [38]. To help healthcare professionals to cope with these stressful situations, some hospitals set up or increased psychological support services, also through online modalities [39], and their effects should be evaluated in the medium and long term [40].

As during the SARS-CoV outbreak in 2002–2004, when 7.6% of the nurses involved in caring patients affected by SARS were looking for another job or considering resignation because of increased work stress and perceived risk of fatality from the unknown virus [9], many nurses during the COVID epidemic expressed fear and inadequacy in caring for patients affected by an emerging infectious disease [33]. Whatever the causative agent is, the COVID-19 pandemic has reminded us all of the paramount importance of protecting the wellbeing of HCWs. Combined with other findings [41], our results might lead health care organizations to prevent a high rate of burnout in future pandemics.

### Limitations

Undoubtedly, the main limitation of this study is the lack of comparison with any previous assessment performed on the hospital staff. Such an initiative never occurred before, and we will leverage the results of this study in order to develop a surveillance program to monitor the psychological sequalae of COVID-19 in front-line HCWs. Among other limitations that may limit the generalizability of the present results, we acknowledge that we did not collect important information about education, socioeconomical status, ethnicity, job satisfaction and marital status that may have increased/decreased the level of burnout in the present sample, thus concealing important adjustments to the final logistic model. In addition, we did not consider the whole staff employed in SICUs, i.e., physicians and physiotherapists, making it impossible to detect any further inter-professional difference.

## 5. Conclusions

Nurses and healthcare assistants operating in SICUs and involved in the care of patients suffering from COVID-19 presented high levels of burnout, particularly emotional exhaustion. The technical care complexity required to stand up to this unprecedented period is probably per se a risk factor for burnout. However, much attention should be devoted to nurses and to young professionals.

Considering the ongoing pandemic, these data suggest the adoption of urgent actions to improve the wellbeing of our HCWs and to set in motion preventive strategies, especially for nurses.

## Figures and Tables

**Table 1:** Characteristics of the healthcare staff working in the SICUs.

Variables	
Age, years	39 (9.8)
Males	40 (38.1)
Females	65 (61.9)
Career, years	
<1	5 (4.8)
1–5	19 (18.1)
6–10	28 (26.7)
≥11	53 (50.5)

Data are presented as mean (SD) or count (%).

**Table 2:** Burnout levels evaluated with Maslach Burnout Inventory for healthcare staff.

MBI Subscales	*n* (%)
Emotional Exhaustion (EE)	
Low	14 (13.3)
Medium	26 (24.8)
High	65 (61.9)
Depersonalization (DP)	
Low	21 (20.0)
Medium	34 (32.4)
High	50 (47.6)
Personal Accomplishment (PA)	
Low	33 (31.4)
Medium	36 (34.3)
High	36 (34.3)

## Data Availability

The data presented in this study are available on request from the corresponding author.
